# A dataset for quantum circuit mapping

**DOI:** 10.1016/j.dib.2021.107526

**Published:** 2021-10-29

**Authors:** Giovanni Acampora, Roberto Schiattarella, Alfredo Troiano

**Affiliations:** a Department of Physics “Ettore Pancini”, University of Naples Federico II, Complesso di Monte Sant’Angelo, Via Cintia 21, Napoli 80126, Italy; b Istituto Nazionale di Fisica Nucleare, Sezione di Napoli, Napoli 80126, Italy; c NetCom Engineering s.p.a., Via Nuova Poggioreale, Napoli 80143, Italy

**Keywords:** Machine learning for quantum computing, Quantum circuit mapping, Quantum computing

## Abstract

Quantum computing is rapidly establishing itself as a new computing paradigm capable of obtaining advantages over its classical counterpart. However, a major limitation in the design of a quantum algorithm is related to the proper mapping of the corresponding circuit to a specific quantum processor so that the underlying physical constraints are satisfied. Moreover, current deterministic mapping algorithms suffer from high run times as the number of qubits to map increases. To bridge the gap in view of the next generation of quantum computers composed of thousands of qubits, this data paper proposes the first datasets that help address the quantum circuit mapping problem as a classification task. Each dataset is composed of random quantum circuits mapped onto a specific IBM quantum processor. In detail, each dataset instance contains some features related to the calibration data of the physical device and others related to the generated quantum circuit. Finally, the instance is labeled with a vector encoding the best mapping among those provided by deterministic mapping algorithms. Considering this, the proposed datasets allow the development of machine learning models capable of achieving mapping similar to those achieved with deterministic algorithms, but in a significantly shorter time.

## Specifications Table

**Table:** 

Subject	Physical sciences
Specific subject area	Quantum computing, Quantum circuit mapping, Quantum compiling
Type of data	CSV files
How data were acquired	The datasets were acquired generating random quantum circuits and mapping them onto IBMQ processors. In detail, each dataset contains set of features related to both the random quantum circuits generated and to the quantum device which each dataset refer. Three datasets are provided, each one refer to a specific IBM quantum machine. Namely, *IBMQ Santiago, IBMQ Athens*, and *IBMQ 16 Melbourne* were the processors used. The target mappings in the datasets were collected selecting the best deterministic mapping among those provided by the algorithms of qiskit transpiler.
Data format	Raw (.csv)
Parameters for data collection	The generated random quantum circuits have a number of qubits belonging to the range [2, processor number of qubits] and their depths vary from 1 to 8. The calibration data for the processors refer to the data provided by IBM daily in the period 31 December 2019–30 June 2021.
Description of data collection	The collection of data can be divided in four steps: **1) Circuit Generation and Date Selection**: a random quantum circuit is generated by Qiskit and simultaneously a date is selected; **2) Circuit Features Extraction**: a set of information related to the generated quantum circuit is extracted; **3) Processor Features Extraction**: the calibration data provided by IBM for the date selected in step 1, are extracted; **4) Label Selection**: the best deterministic mapping provided by qiskit transpiler is selected as label of the instance.
Data source location	Quantum Computing and Smart Systems (QUASAR) Laboratory, University of Naples Federico II, Naples, Italy
Data accessibility	Schiattarella, Roberto; Acampora, Giovanni (2021), “Dataset for Quantum Circuits Mapping”, Mendeley Data, V1, doi: 10.17632/pmycgb2bt7.1
Related research article	Giovanni Acampora and Roberto Schiattarella. Deep neural networks for quantum circuit mapping. Neural Computing and Applications, pages 1–21, 2021. https://doi.org/10.1007/s00521-021-06009-3

## Value of the Data

• The proposed datasets can be helpful to address the Quantum Circuit Mapping on NISQ processors problem as multi-output classification task. This can significantly reduce the execution time of current mapping algorithms, speeding up the whole quantum compiling process in view of the next generation of quantum processors. The proposed datasets are the firsts in literature.
• Reducing the computational time of the quantum circuit mapping is a key point for the development of fully efficient quantum devices. Therefore these datasets can be used by researchers to develop innovative circuit mapping techniques based on machine-learning and artificial intelligence.
• Considering this, the datasets lend themselves to any multi-output classification algorithm. Furthermore, given the high dimensionality of the proposed data, techniques of dimensionality reduction can also be used and tested on it.
• These data were acquired as proposed in Acampora and Schiattarella [1] where a Deep Neural Network was used to perform the multi-output classification task.

## 1 Data Description

Besides the issues related to their size and noise, a critical problem that characterizes the current NISQ devices (Noisy Intermediate-Scale Quantum) [2] is the low connectivity of their *coupling maps*, for which each qubit is connected to a limited number of other qubits. Considering this, there is a strong demand for quantum compilers able to identify efficient initial mapping among circuit qubits and processor qubits, so as to optimize in a reasonable amount of time the number of SWAP operations required to execute the compiled circuit. To bridge this gap this paper proposes the firsts dataset useful to address the quantum circuit mapping problem as classification task. The provided datesets are csv files named as the IBM quantum processor which they refer. Considering a quantum circuit $C=\{q_{1}^{C},\ldots ,q_{N}^{C}\}$ composed of N qubits, and a quantum processor $P=\{q_{1},\ldots ,q_{M}\}$ with M qubits, then the information related to C in each dataset can be summarized as follows:

• *N* - an integer value representing the number of circuit qubits. In the csv files this feature is contained in the column named $N_{qubits}$. The values of N for each proposed dataset range from 2 to M, because 2 is the minimal number of qubits required to build quantum circuits composed of multi-qubits gates, while M is the maximal number of circuit qubits that can be mapped onto a M qubits quantum processor;
• $N_{cx}$ - an integer value representing the total number of CNOT gates in the circuit C. The range of values of $N_{cx}$ varies for each of the proposed dataset and it depends on the random quantum circuits considered during the data acquisition procedure. For the dataset related to *Athens*, $N_{cx}$ ranges from 0 to 69, for the one related to the *Santiago* quantum processor it varies from 0 to 71 and finally, for the dataset related to the *Melbourne* device it ranges from 0 to 113;
• $N_{measures}$ - an integer value representing the number of measurement operations in the circuit C. In the csv files this number is contained in the column $N_{measures}$. Moreover, because all the random quantum circuits considered to collect the data end with a measurement operation on each circuit qubit, the number $N_{measures}$ is always equal to N;
• $N_{i,j}^{cx}$ - a matrix of integer values where the item [i,j] contains the number of CNOT gates between the control qubits $q_{i}^{C}$ and the target qubit $q_{j}^{C}$ of the circuit C. Each element of $N_{i,j}^{cx}$ is reported in the datasets in the column cx_i_j. As for $N_{cx}$, the range of values for each element of $N_{i,j}^{cx}$ is strictly related to the random quantum circuits obtained during the data acquisition procedure. However, it is always true that $N_{i,j}^{cx}\geq 0$ and $\sum_{i,j}N_{i,j}^{cx}=N_{cx}$.

Similarly, with respect to the processor P, the following information have been considered:

• Date of calibration data- a date related to when the calibration data of P refer. Column last_update_date in the datasets. The calibration data refer to random day in the period 31 December 2019-30 June 2021;
• $CX_{i,j}^{ER}$ - an array of real values where each value represents the error rate of a CNOT using $q_{i}$ as control qubit and $q_{j}$ as target qubit for each $(q_{i},q_{j})\in P$. If $\left(q_{i},q_{j}\right)$ are not connected in the processor’s coupling map there is a default value set to 100,000. Both for the devices of *Athens, Santiago* and *Melbourne* these quantities are symmetrical, i.e. $CX_{i,j}^{ER}=CX_{j,i}^{ER}$. These values are limited between 0 and 1. In the csv files each element $CX_{i,j}^{ER}$ is inserted in the column named edge_error_i_j;
• $CX_{i,j}^{ET}$ - an array of real values where each value represents the execution time (in nanoseconds) of a CNOT gate using $q_{i}$ as control qubit and $q_{j}$ as target qubit for each $(q_{i},q_{j})\in P$. If $\left(q_{i},q_{j}\right)$ are not connected in the processor’s coupling map there is a default value set to 100,000. In contrast to the values for error rates, the gate times are not symmetrical, i.e. $CX_{i,j}^{ET}\neq CX_{j,i}^{ET}$. In the csv files each element $CX_{i,j}^{ET}$ is inserted in the column named edge_length_i_j;
• $T_{1}$ - an array of real values where each value represents the longitudinal relaxation time ($T1_{i}$) in micro-seconds characterizing a qubit $q_{i}$ of the processor P. Each element of $T_{1}$ is reported in the $T1_{i}$ column of the datasets;
• $T_{2}$ - an array of real values where each value represents the transverse relaxation time in micro-seconds ($T2_{i}$) characterizing a qubit $q_{i}$ of the processor P. Each element of $T_{2}$ is reported in the $T2_{i}$ column of the datasets;
• $E^{R0}$ an array of real values where each value $E_{i}^{R0}$ represents the readout error characterizing a qubit $q_{i}$ of the processor P. A readout error can assume values ranging from 0 to 1. Each $E_{i}^{R0}$ is inserted in the column readout_error_i of the datasets.

A schematic view of the features related to the quantum circuit mapping problem is provided in Table 2 in Acampora and Schiattarella [1].

Finally, each instance of the dataset is labelled as follows:

• Label - an *M*-dimensional array of integers, where the position of each value refer to processor qubit index and the integer value refer to the circuit qubit index mapped on it. In the csv files, the label vector is contained in the final *M* columns of each file. Each of this column is named with an integer that represent the related index of the processor qubit.

If M>N, a NaN value is present in the columns related to processor qubits not involved during the mapping operation.

**Fig. 1:**
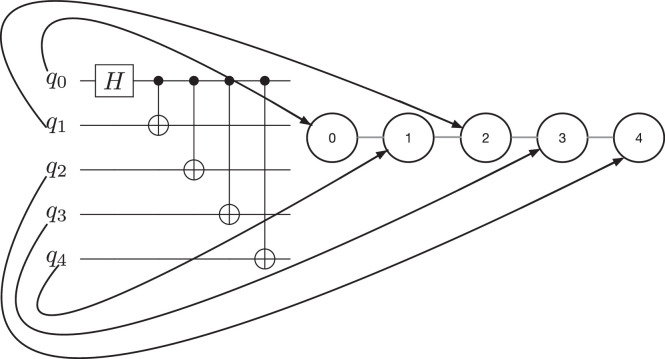
Example of circuit mapping.

To better understand how the mapping is encoded in the dataset label let us analyze the example in Fig. 1: a 5-qubits quantum circuit (on the left) is mapped onto a 5-qubits quantum processor (on the right). The black arrows in the figure represent the mapping performed. For a five qubits processor, the label in the related dataset is an array of five elements and the mapping in Fig. 1 is encoded in it as follows:

(1) Label=[0,4,1,3,2]

**Fig. 2:**
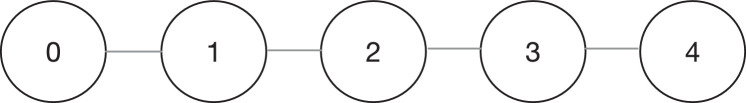
*ibmq_santiago* and *ibmq_athens* coupling map.
• The **Athens.csv** file which contains random quantum circuits composed from two to five qubits mapped on *ibmq_athens* quantum processor. This device is characterized by a Quantum Volume of 32, 5 physical qubits and from the coupling map reported in Fig. 2. The dataset contains 66,747 random quantum circuits with number of CX which varies from 0 to 69. In detail, the file contains 4660 2-qubits circuits, 16,201 3-qubits circuits, 19,128 4-qubits circuits and 26,758 5-qubits circuits. Overall, the dataset is composed of 84 columns.
• The **Melbourne.csv** file which contains random quantum circuits composed from two to fifteen qubits mapped on *ibmq_16_melbourne* quantum processor. This device is characterized by a Quantum Volume of 8, 15 physical qubits and from the coupling map reported in Fig. 3. The dataset contains 47,111 random quantum circuits with number of CX which varies from 0 to 113. In detail, the file contains 1330 2-qubits circuits, 3355 3-qubits circuits, 3595 4-qubits circuits, 3644 5-qubits circuits, 3634 6-qubits circuits, 3590 7-qubits circuits, 3571 8-qubits circuits, 3553 9-qubits circuits, 3537 10-qubits circuits, 3513 11-qubits circuits, 3488 12-qubits circuits, 3455 13-qubits circuits, 3427 14-qubits circuits and 3419 15-qubits circuits. Overall, the dataset is composed of 694 columns.

In detail, the provided files are the following:

• The **Santiago.csv** file which contains random quantum circuits composed from two to five qubits mapped on *ibmq_santiago* quantum processor. This device is characterized by a Quantum Volume of 32, 5 physical qubits and from the coupling map reported in Fig. 2. The dataset contains 74,576 random quantum circuits with number of CX which varies from 0 to 71. In detail, the file contains 4862 2-qubits circuits, 19,197 3-qubits circuits, 21,552 4-qubits circuits and 28,965 5-qubits circuits. Overall, the dataset is composed of 84 columns.

**Fig. 3:**
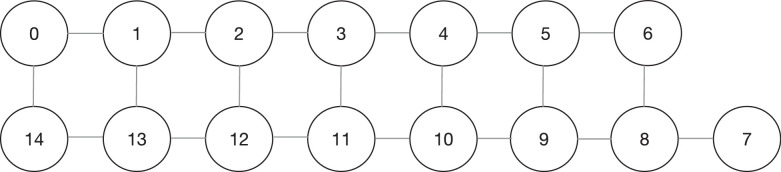
*ibmq_16_melbourne* coupling map.

## 2 Experimental Design, Materials and Methods

In this section the data acquisition procedure is discussed. All the dataset have been collected using Python^1^: in detail, for each of them, an empty pandas dataframe was filled in with the procedure hereafter described. Such process makes intensive use of the Qiskit^2^ library [3]. The steps to insert a row in the dataframe are now analysed:

1. Generation of a random quantum circuit using the generator provided by Qiskit: in detail, this step is useful to create random quantum circuits composed of single and multi-qubits logical gates. The number of qubits in the circuit ranges from 2 to the number of qubits composing the quantum processor. The initial depth of the generated circuit is a value selected in the range 1–8. Furthermore, the quantum circuit ends with a measurement gate for each qubits. Once the quantum circuit is obtained, the quantum gates that compose it are unrolled in terms of the following basis gates:
  (2) $$\mathrm{BG}=\left[U_{1},U_{2},U_{3},CX,ID\right]$$
  using the Unroll^3^ pass available in the traspiling methods of Qiskit. In Eq. (2) ID indicates the identical operator, CX is the controlled-not gate, $U_{3}$ represents the most general single-qubit quantum gate, whose matrix form is given in Eq. (3), whereas $U_{2}$ and $U_{1}$ are respectively obtained from $U_{3}$ setting $\theta =\frac{\pi }{2}$ and θ=ϕ=0.
  (3) $$U_{3}\left(\theta ,\phi ,\lambda \right)=\left(\begin{array}{cc} \cos(\frac{\theta }{2}) & -e^{i\lambda }\sin\left(\frac{\theta }{2}\right)\\ e^{i\phi }\sin\left(\frac{\theta }{2}\right) & e^{i\lambda +i\phi }\cos\left(\frac{\theta }{2}\right) \end{array}\right)$$
  At this point, the number of circuit qubits and the number of measurement operations are inserted in the dataframe;
2. Extrapolation of Quantum Circuit Features: considering the operations performed in the previous step, the unrolled quantum circuit is composed of single-qubit quantum gates $ID,U_{1},U_{2}$ and $U_{3}$ and only CX gates as multi-qubits gates. At this point, the total number of controlled not quantum gates $N_{cx}$ is collected together with the matrix $N^{cx}$, whose item [i,j] contains the number of CX gates between the *i*th and the *j*th qubit of the circuit where the further is the control qubit and the latter the target one. $N_{cx}$ and $N_{i,j}^{cx}$ are inserted in the dataframe;
3. Extrapolation of Quantum Processor Features: in this step a random date is selected (the datasets are limited to the period 31 December 2019–30 June 2021) and the calibration data provided from IBM for that day are retrieved. If for some reasons the calibration data are unavailable for the selected date, a new random day is selected. The calibration data contains all the features related to the processor *P* described in previous section, that are therefore collected and inserted in the pandas dataframe;
4. Selection of the best deterministic circuit mapping among those computed by using well-known algorithms available in IBM Qiskit, namely *Dense Layout, Noise Adaptive Layout* [4] and *SABRE Layout* [5]: In this context, the best mapping of a circuit is the one that generates a new circuit characterized by the smaller number of SWAP gates. The number of SWAP gates in a circuit is computed by means of three IBM Qiskit routing algorithms, named *Lookahead Swap*^4^, *Stochastic Swap*^5^ and *Sabre Swap*.^6^
  Therefore, the process of labeling each circuit c randomly generated is outlined as follows:
  4.1 Compute the initial mappings for the circuit c by using both *Dense Layout* and *SABRE Layout* and *Noise Adaptive Layout* approaches;
  4.2 For each mapping computed at previous step, compute the number of SWAPs needed to run the circuit c by using both *Lookahead Swap* and *Sabre Swap* and *Stochastic Swap* approaches;
  4.3 Choose the mapping requiring the smaller number of SWAP gate executions.
  The mapping is encoded in a vector whose index represents the index of the processor qubits and whose elements the index of the circuit qubits. See Eq. (1) for a practical example.
  At this point the vector is inserted in the dataframe row, which is now completed.

The row is saved in a csv file and a new iteration of steps 1–4 is carried out to build a new dataset line. Finally, each obtained dataset have been cleared from duplicate instances using the pandas^7^ drop_duplicate method.

## Ethics Statement

The authors declare that this work does not involve the use of human subjects or experimentation with animals.

## CRediT authorship contribution statement

**Giovanni Acampora:** Supervision, Conceptualization, Methodology, Writing – original draft. **Roberto Schiattarella:** Conceptualization, Software, Writing – original draft, Validation, Formal analysis, Investigation. **Alfredo Troiano:** Writing – original draft.

## Declaration of Competing Interest

The authors declare that they have no known competing financial interests or personal relationships which have, or could be perceived to have, influenced the work reported in this article.

## Appendix Supplementary materials

Image, application 1

## Footnotes

1 Python 3.7.6.

2 The versions of qiskit used to collect the data are qiskit 0.23.1.; qiskit-terra 0.16.1; qiskit-ibmq-provider 0.11.1; qiskit-aer 0.7.1.

7 https://pandas.pydata.org.

## Footnotes

3 Unroller.

4 IBM Qiskit Lookahead Swap Algorithm.

5 IBM Qiskit Stochastic Swap Algorithm.

6 IBM Qiskit Sabre Swap Algorithm.
