# Supplementary figures and images for: A dataset for quantum circuit mapping
Source: Data Brief. 2021 Oct 29;39:107526. doi: 10.1016/j.dib.2021.107526 (PMC8581508; doi:10.1016/j.dib.2021.107526)

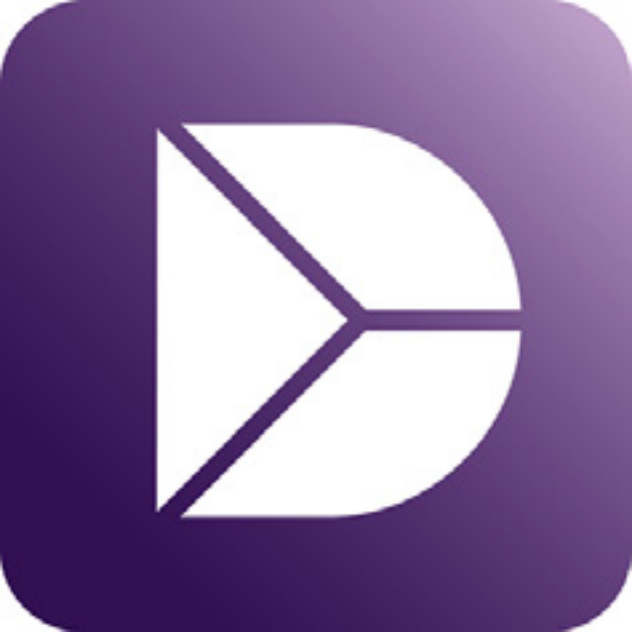

Supplement: Supplementary file 1 [file mmc1.zip › dib-logo.pdf]

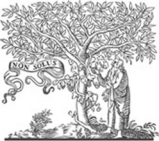

ELSEVIER

Supplement: Supplementary file 1 [file mmc1.zip › elsevier-logo.pdf]
